# Prevalence of rotator cuff tear concomitant with neuropathy: analysis of 659 cases using needle electromyography

**DOI:** 10.1016/j.xrrt.2026.100762

**Published:** 2026-04-30

**Authors:** Nobuyasu Ochiai, Eiko Hashimoto, Seiji Ohtori, Norimasa Takahashi, Keisuke Matsuki, Hiroyuki Sugaya

**Affiliations:** a Department of Orthopaedic Surgery, Chiba University, Chiba, Japan; b Funabashi Orthopaedic Hospital, Funabashi, Japan; c Tokyo Sports & Orthopaedic Clinic (TSOC), Tokyo, Japan

**Keywords:** Rotator cuff tear, Suprascapular neuropathy, Needle electromyography, Psuedoparalysis, MRI, Neuropathy

## Abstract

**Background:**

Rotator cuff tears (RCTs) are highly prevalent and increase with age. Although suprascapular neuropathy associated with RCTs has been increasingly reported, the prevalence of other neuropathies, including cervical radiculopathy and brachial plexopathy, remains unclear. Furthermore, the relationship among neuropathy, tear size, and shoulder pseudoparalysis remains incompletely understood. This study aimed to determine the prevalence of neuropathies associated with RCTs using needle electromyography (EMG) and examine their relationship with tear size and pseudoparalysis.

**Methods:**

A total of 659 patients with surgically treated magnetic resonance imaging–confirmed RCTs between 2012 and 2014 were included in the study. All patients underwent pre-operative needle EMG. Neuropathy was classified as suprascapular neuropathy, axillary nerve palsy, or proximal neuropathy (cervical radiculopathy or brachial plexopathy). Tear size was categorized as subscapularis, partial thickness, small, medium, large, or massive. Pseudoparalysis was defined as active shoulder elevation ≤90°. Statistical analyses included one-way analysis of variance and chi-square tests with post-hoc residual analyses. Statistical significance was set at *P* < .05.

**Results:**

Of the 659 patients, 105 (15.9%) had abnormal EMG findings, including 48 (7.3%) with suprascapular neuropathy, 3 (0.5%) with axillary nerve palsy, and 54 (8.2%) with proximal neuropathy. The prevalence of neuropathy increased with tear size and was the highest in the massive tear group, where proximal neuropathy was present in 40.4% of cases. Nerve status was significantly associated with tear size (*P* < .001), with suprascapular and proximal neuropathy being significantly over-represented in massive tears. Patients with proximal neuropathy were significantly older than those without proximal neuropathy (*P* = .0001). Shoulder pseudoparalysis occurred in 91 patients (13.8%). Among these, proximal neuropathy was the most common abnormality, identified in 42.9%. The prevalence of pseudoparalysis increased with tear size and was the highest in the massive tear group. A significant association was observed between neuropathy and pseudoparalysis (*P* < .05).

**Conclusion:**

Neuropathy, particularly proximal neuropathy, was strongly associated with larger RCTs and shoulder pseudoparalysis. These findings suggest that neurological involvement is not incidental but may contribute to disease severity, functional impairment, and the progression of fatty infiltration through the combined effects of disuse and neuropathy. Needle EMG is a valuable tool for detecting neuropathies and may improve diagnostic accuracy and treatment planning. Therefore, EMG should be considered selectively, particularly in patients with large-to-massive tears, severe muscle atrophy, or pseudoparalysis rather than being routinely performed in all cases.

Rotator cuff tears (RCTs) are among the most common orthopedic shoulder disorders.^20^^,^^22^ The prevalence of symptomatic and asymptomatic RCTs increases with age.^22^ Recently, suprascapular neuropathy associated with RCTs has been increasingly reported, with growing interest in this entity over the past decade.^6^^,^^7^^,^^13^^,^^14^^,^^21^ Furthermore, suprascapular neuropathy secondary to RCTs is thought to result from traction or compression of the suprascapular nerve in massive RCTs.^13^ Vad et al reported that suprascapular neuropathy was associated with 8% (2) of 25 massive RCTs with muscle atrophy,^21^ Mallon et al reported that all 8 individuals with massive RCTs (>5 cm) in their study had fatty infiltration of the muscle,^14^ and Costouros et al reported that 27% (7) of 26 patients with massive RCTs and fatty infiltration had suprascapular neuropathy.^7^ Collin et al reported that of 49 shoulders, 6 (12%) had neurologic lesions noted on electromyography (EMG): one suprascapular nerve neuropathy, one radicular lesion of the C5 root, one affected electromyogram in the context of a previous stroke, and 3 cases of partial axillary nerve palsy with a history of shoulder dislocation. They concluded that the prevalence of suprascapular neuropathy in massive RCTs was low.^6^

RCTs and shoulder neuropathies may have similar clinical presentations. Pain, shoulder muscle atrophy, and limitations in motion are common features of both disorders. It is often difficult to distinguish between these entities using medical history, physical examination, and EMG. When they coexist, diagnosis becomes even more challenging because the symptoms of neuropathy may be obscured by the RCTs itself.^5^ Magnetic resonance imaging (MRI) reveals full-thickness RCTs that account for muscle weakness and atrophy, while obscuring the clinical features of concomitant neuropathies. Experimental evidence from rat models demonstrates that concomitant neuropathy proximal to the suprascapular nerve adversely affects both the structural integrity and healing capacity of the rotator cuff. When brachial plexus or cervical-level nerve injury is present, rotator cuff tendons exhibit increased degeneration, inferior biomechanical properties, and impaired healing after repair, resulting in increased susceptibility to post-operative retear. These findings support the concept that neuropathy proximal to the suprascapular nerve is an independent risk factor for rotator cuff failure, particularly in massive tears characterized by advanced fatty degeneration.^1^^,^^19^ Therefore, identification of neuropathy before rotator cuff surgery is clinically important. Vad et al reported that there was a 28% prevalence of associated peripheral neuropathy, including upper trunk brachial plexopathy and cervical radiculopathy, and also found that greater degrees of atrophy were significantly associated with the presence of neuropathy.^21^ However, that study included only 25 patients; therefore, the true prevalence of neuropathies associated with RCTs remains unclear. Based on these findings, we hypothesized that electrodiagnostic abnormalities detected on needle EMG would be more common in advanced RCTs, particularly in large-to-massive tears and in cases of pseudoparalysis, even in the absence of clinically overt neuropathy. This study aimed to determine the prevalence of suprascapular neuropathy, cervical spine lesions, brachial plexopathy, and other neuropathies associated with RCTs using needle EMG.

## Materials and methods

After obtaining approval from our Institutional Review Board, patients with RCTs confirmed by MRI were included in this study. All patients underwent needle EMG (Neuropack S1; Nihon Kohden, Tokyo, Japan) before surgery performed by a single orthopedic doctor (N.O.) with formal training in EMG. The treating surgeons (H.S. and N.T.) were not involved in the EMG examinations and were blinded to the EMG findings at the time of clinical evaluation. All patients underwent arthroscopic rotator cuff repair or rTSA between 2012 and 2014. A total of 659 patients were included. The average age of the patients was 65.4 ± 9.1 years (range: 37-90). The study included 336 male and 323 female patients. The tear sizes were as follows: isolated subscapularis (SSC) tear, 3 cases (average age: 73.3 ± 3.1 years; male: 2 patients; female: 1 patient); partial thickness (PT) RCTs, 53 cases (average age: 59.2 ± 10.1 years; male: 27 patients; female: 26 patients); small (SC) RCTs (<1 cm), 112 cases (average age: 63.3 ± 9.4 years; male: 57 patients; female: 55 patients); medium-sized (MC) RCTs (1-3 cm), 203 cases (average age: 65.3 ± 8.7 years; male: 98 patients; female: 105 patients); large (LC) RCTs (3-5 cm), 184 cases (average age: 66.2 ± 8.2 years; male: 95 patients; female: 89 patients); massive RCTs (>5 cm), 104 cases (average age: 69.5 ± 8.6 years; male: 57 patients; female: 47 patients). Tear size was confirmed arthroscopically or macroscopically during the surgery. Among these patients, 3 had a history of shoulder dislocation.

EMG was performed the day before surgery. EMG was performed on the rhomboid (mainly innervated at the C4 level), supraspinatus, infraspinatus, deltoid, biceps (mainly innervated at the C5 and 6 levels), extensor digitorum communis (EDC) (mainly innervated at the C7 level), and abductor digiti quinti (mainly innervated at the C8 level). If a fibrillation potential, positive sharp wave, polyphasic wave, or giant spike was found during EMG, the patient was diagnosed with neuropathy. If abnormal waves were found only in the supraspinatus or infraspinatus, the patient was diagnosed with suprascapular nerve neuropathy; if abnormal waves were found only in the deltoid, the patient was diagnosed with axillary nerve palsy. When abnormalities extended beyond the supraspinatus or infraspinatus to other muscles, patients were classified as having cervical radiculopathy or brachial plexopathy, indicating a lesion proximal to the branching of the suprascapular and axillary nerves from the brachial plexus. Specifically, abnormalities involving the biceps were classified as C5-C6 neuropathy, whereas abnormalities involving the rhomboid, biceps, EDC, and abductor digiti quinti were classified as C4-C8 neuropathy. Proximal neuropathy was diagnosed only when abnormalities were found in multiple muscle groups beyond the suprascapular nerve or axillary nerve distribution, consistent with cervical radiculopathy or brachial plexopathy (proximal neuropathy groups). If abnormalities were found only in the biceps, EDC, and abductor digit quinti or in a combination limited to these 3 muscles, the case was not diagnosed as proximal neuropathy.

Furthermore, all patients were assessed for the presence of a pseudoparalytic shoulder, defined as active shoulder elevation of 90° or less despite pain-relieving measures, including injection, administered in the outpatient clinic before admission.^3^ The ability to achieve active elevation greater than 90° was evaluated by 2 surgeons (H.S. and N.T.).

### Statistical analysis

All statistical analyses were performed using JMP Pro 18.0 software (SAS Institute Inc., Cary, North Carolina, USA). First, age distribution across RCT sizes and types of neuropathy was compared. For continuous variables (age), differences among multiple groups were analyzed using one-way analysis of variance. When a significant overall difference was detected, post-hoc pairwise comparisons were conducted to identify specific group differences. For categorical variables, including the relationships between neuropathy type and tear size, as well as the presence of neuropathy and pseudoparalysis, contingency table analyses were performed using the chi-square test. Where appropriate, post-hoc analyses based on adjusted residuals were performed to identify specific group differences. Standardized residuals more than +1.96 or less than −1.96 were considered to indicate a statistically significant deviation from the expected frequency. A *P* value of < .05 was used to determine statistical significance.

## Results

**Figure 1:**
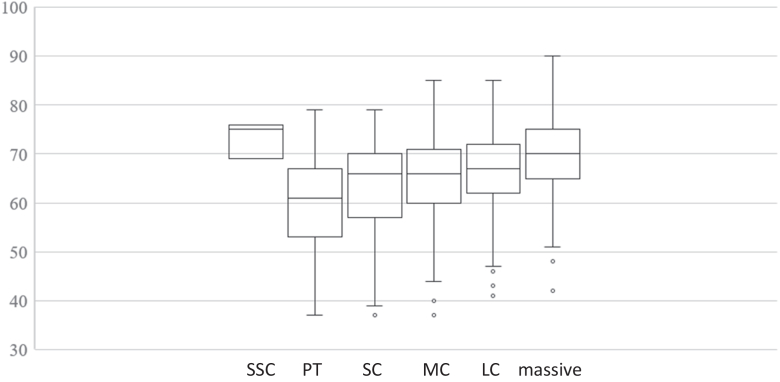
Comparison of patient age according to rotator cuff tear size. Tear sizes are categorized as subscapularis (SSC), partial thickness (PT), small (SC), medium (MC), large (LC), and massive tears. Significant differences in age are observed among groups.

First, in the comparison of age across tear-size groups, the PT group was significantly younger than the MC (*P* = .0032), LC (*P* = .0001), and massive tear groups (*P* < .0001), whereas the massive tear group was significantly older than the SC (*P* < .0001), MC (*P* = .0004), and LC groups (*P* = .0108) (Fig. 1).

**Figure 2:**
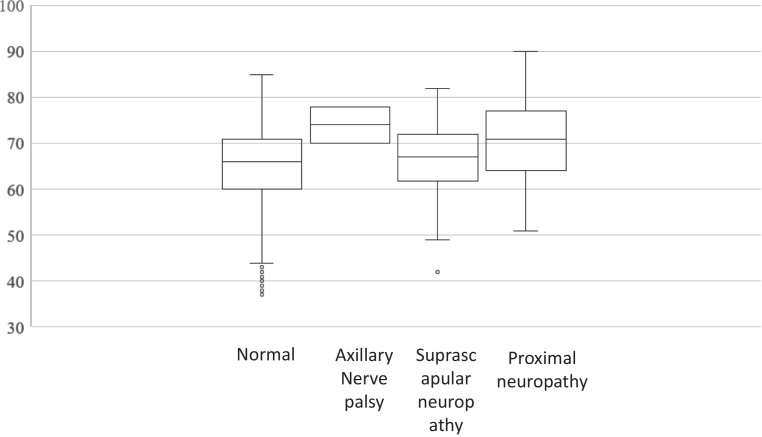
Comparison of patient age according to neuropathy types. Patients are categorized as normal, axillary nerve palsy, suprascapular neuropathy, or proximal neuropathy.

**Figure 3:**
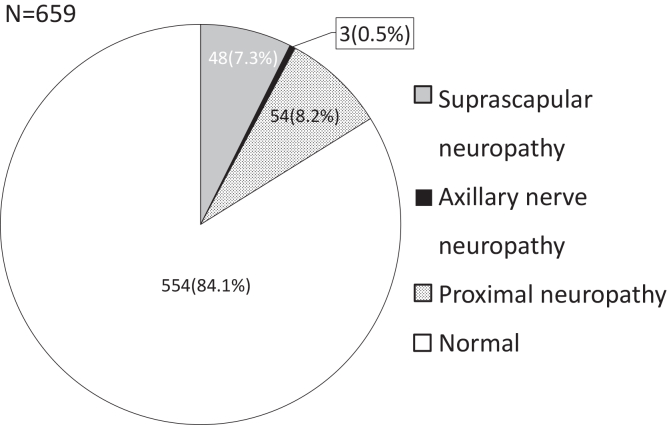
Distribution of electromyography (EMG) findings in the study population (N = 659). Patients are classified into 4 groups: normal, axillary nerve palsy, suprascapular neuropathy, and proximal neuropathy.

Among the 659 patients, normal EMG was present in 554 patients (84.1%; mean age: 64.2 ± 10.4 years; 275 male and 279 female patients). Isolated suprascapular neuropathy was found in 48 cases (7.3%; mean age: 66.3 ± 7.8 years; 25 male and 23 female patients). Axillary nerve palsy due to the dislocation of the shoulder was found in 3 cases (0.5%; average age: 74.0 ± 8.4 years; male: 1 patient; female: 2 patients). In contrast, 54 cases were diagnosed as proximal neuropathy (8.2%; mean age: 70.8 ± 8.6 years; 35 male and 19 female patients) (Figs. 2 and 3). When comparing types of neuropathy and age, the proximal neuropathy group was significantly older than both the normal group (*P* = .0001) and the suprascapular neuropathy group (*P* = .0321). No neuropathy was observed in the SSC and PT groups. One case of proximal neuropathy was observed in the SC group (1/112; 0.9%). In the MC group, 3 cases of suprascapular neuropathy (3/203 cases; 1.5%), one case of axillary nerve palsy due to dislocation of the shoulder (1/203; 0.5%), and 4 cases of proximal neuropathy were found (4/203; 2.0%). These results suggest that the prevalence of neuropathy was lower in patients with smaller tears. By contrast, 21 cases of suprascapular neuropathy (21/184; 11.4%), one case of axillary nerve palsy due to dislocation of the shoulder (1/184; 0.5%), and 7 cases of proximal neuropathy were found (7/184; 3.8%) in the LC group. Furthermore, 24 cases of suprascapular neuropathy (24/104; 23.1%), one case of axillary nerve palsy due to dislocation of the shoulder (1/104; 1%), and 42 cases of proximal neuropathy were found (42/104; 40.4%) in the massive group. Only 35.6% of patients in the massive-tear group were free of neuropathy (Table I). A significant association was observed between nerve status and tear size (chi-square test, *P* < .001). Residual analysis demonstrated a nonuniform distribution of nerve types across tear sizes. Specifically, the massive group showed a marked over-representation of suprascapular neuropathy and proximal neuropathy (standardized residuals: +6.60 and +10.46, respectively), whereas these nerve types were significantly under-represented in smaller tear sizes (PT, SC, and MC groups). Conversely, normal EMG findings were significantly under-represented in the massive group (standardized residual: −5.25).

**Table I:** Prevalence of neuropathy according to tear size.

Size and location of rotator cuff tear	Normal (%)	Suprascapularneuropathy (%)	Axialnervepalsy (%)	Proximalneuropathy(%)
SSC	100	0	0	0
PT	100	0	0	0
SC	99.1	0	0	0.9
MC	96.1	1.5	0.5	2.0
LC	84.2	11.4	0.5	3.8
Massive	35.6	23.1	1.0	40.4

*SSC*, subscapularis; *PT*, partial thickness; *SC*, small; *MC*, medium; *LC*, large.

**Figure 4:**
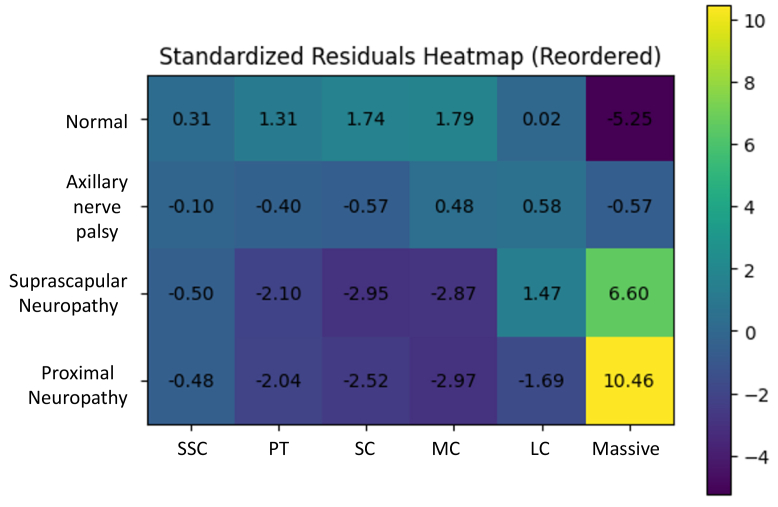
Heatmap of standardized residuals illustrating the association between nerve status and rotator cuff tear size derived from chi-square analysis. *SSC*, subscapularis; *PT*, partial thickness; *SC*, small; *MC*, medium; *LC*, large.

These findings indicate that specific nerve types are disproportionately associated with larger tears, contributing substantially to the overall association (Fig. 4).

**Figure 5:**
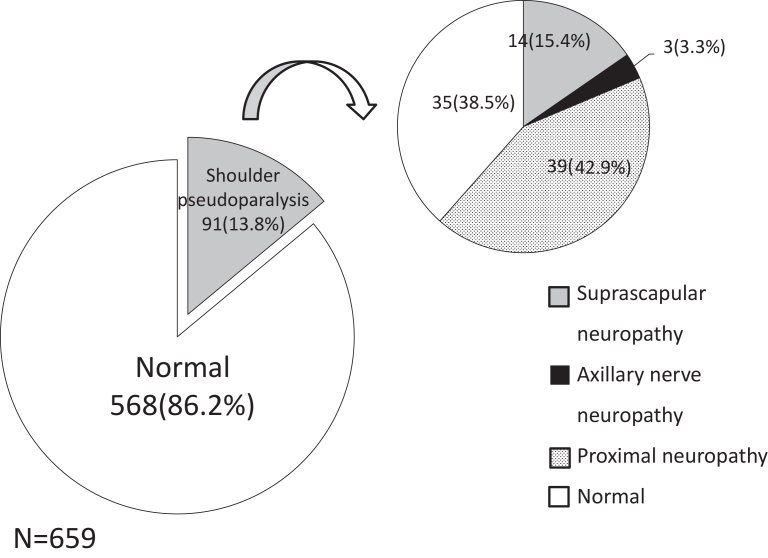
Distribution of shoulder pseudoparalysis in the study population (N = 659) and distribution of nerve status among shoulder pseudoparalysis.

Shoulder pseudoparalysis was identified in 91 of 659 patients (91/659; 13.8%) (Fig. 5). Among the 91 patients, 14 cases were diagnosed with suprascapular neuropathy (14/91; 15.4%), 3 cases were axillary nerve palsy (3/91; 3.3%), and 39 cases were diagnosed as proximal neuropathy (39/91; 42.9%). No shoulder pseudoparalysis was observed in the SSC, PT, or SC groups according to tear size. There were 6 cases of shoulder pseudoparalysis in the MC group. Among the 6 patients, 3 were normal, one was diagnosed with axillary nerve palsy, and 2 were diagnosed with proximal neuropathy. There were 32 cases of shoulder pseudoparalysis in the LC group. Among these 32 patients, 21 were normal, 5 were diagnosed with suprascapular neuropathy, one was diagnosed with axillary nerve palsy, and 5 were diagnosed with proximal neuropathy. There were 53 cases of shoulder pseudoparalysis in the massive group. Among 53 cases, only 11 cases were normal and 9 cases were diagnosed as suprascapular neuropathy (17.0%) and 1 case was diagnosed as axillary nerve palsy (1.9%) and 32 cases of 53 cases (60.3%) were diagnosed as proximal neuropathy. Furthermore, a significant association was observed between the presence of neuropathy and pseudoparalysis (chi-square test, *P* < .05). Patients with neuropathy showed a higher prevalence of pseudoparalysis compared with those without neuropathy.

## Discussion

The most important finding of this study was the strong association of neuropathy, particularly proximal neuropathy, with increasing RCT size and shoulder pseudoparalysis. In this cohort of 659 patients, the prevalence of neuropathy increased progressively with tear size and was most pronounced in the massive tear group. Residual analysis further demonstrated that suprascapular neuropathy and proximal neuropathy were significantly over-represented in massive tears, whereas normal nerve status was under-represented. These findings suggest that neurologic involvement is not incidental but is closely linked to the severity of rotator cuff pathology.

In addition, patients with proximal neuropathy were significantly older than those without neuropathy or with isolated suprascapular neuropathy, indicating that age-related factors may contribute to more extensive neural involvement. Importantly, neuropathy was also significantly associated with shoulder pseudoparalysis. Among patients with pseudoparalysis, proximal neuropathy was the most prevalent nerve disorder, particularly in the massive tear group, where it accounted for the majority of cases. This supports the concept that neural dysfunction, in addition to structural tendon damage, plays a critical role in severe functional impairment. Overall, these results indicate that both tear size and neurologic status should be considered when evaluating patients with RCTs, as neuropathy may substantially influence clinical presentation and functional outcomes.

The prevalence of RCTs increases with age, and the number of tears increases over time.^16^^,^^20^ Although cervical spondylosis is age-related, cervical radiculopathy is most prevalent in the sixth decade of life.^18^ Similar to RCTs, cervical radiculopathy can cause shoulder pain, weakness, and muscle atrophy. Given the high prevalence of RCTs with age, it is reasonable to assume that they may coexist with cervical radiculopathy as both conditions become more common with aging. If RCTs and cervical radiculopathy coexist, their clinical manifestations may overlap considerably. Both disorders may contribute to progressive fatty infiltration. Our results support and extend previous concepts by suggesting that age-related neuropathic changes, including cervical or proximal nerve involvement, may coexist with and potentially exacerbate rotator cuff pathology. The overlap in clinical presentation and the shared association with fatty degeneration imply that neuropathy is not merely a coincidental finding but may play a contributory role in tear progression and functional deterioration.

Although fatty degeneration of the rotator cuff muscles progresses over time,^8^, ^9^, ^10^ its etiology remains unclear. Recently, there have been several reports of massive RCTs concomitant with suprascapular neuropathy, which is thought to be one of the factors contributing to worsening fatty infiltration.^7^^,^^14^ Although the prevalence of suprascapular neuropathy increases with increasing tear size, it is necessary to rule out other neuropathies, such as cervical radiculopathy. Hattrup et al emphasized that appropriate diagnosis is important for planning proper treatment.^11^ The diagnosis of an RCT can be made with physical examinations, neurologic examinations, and imaging studies such as MRI. Electrophysiological studies can be useful in confirming the diagnosis and excluding other neurological conditions.^4^^,^^15^ Furthermore, neurophysiological testing is particularly helpful when clinical and imaging examinations are inconclusive.^2^ EMG studies can be confirmatory and have been reported to detect muscle denervation with 91% accuracy.^17^ To distinguish suprascapular neuropathy from other neuropathies, it is necessary to examine other muscles, such as the deltoid, biceps, and rhomboid muscles to exclude cervical radiculopathy or brachial plexopathy. Although many studies have reported suprascapular neuropathy, there are only 2 studies on the incidence of musculoskeletal shoulder disorders concomitant with other neurological conditions. Vad et al reported that among 25 patients diagnosed using EMG, 28% had neurological conditions such as upper trunk or axillary neuropathy, suprascapular neuropathy, and cervical radiculopathy; the subjects were too small.^21^ Hawkins et al reported 13 cases; however, their study included only a small number of RCTs.^12^ Based on these results, it was impossible to determine the prevalence of neuropathy concomitant with RCTs. In contrast, our data represent the first large-scale study of neuropathy associated with RCTs. In our cohort, 40.4% of patients in the massive-tear group had proximal neuropathy. Furthermore, only 35.6% of patients in the massive-tear group were free of neuropathy. These results suggest that larger tear size is associated with a greater risk of concomitant neuropathy. Furthermore, our data showed that the prevalence of proximal neuropathy increased in patients with shoulder pseudoparalysis, particularly in those with large or massive tears. If RCTs coexist with neuropathy, fatty infiltration worsens because of disuse atrophy due to the RCTs and atrophy due to neuropathy. Although EMG is not routinely performed in patients with RCTs, it should be considered to perform EMG to exclude neuropathy in cases of severe atrophy of the rotator cuff muscles or large tears.

Our study had some limitations. First, we did not perform radiography or MRI of the cervical spine in all patients; therefore, it was difficult to accurately distinguish cervical radiculopathy from brachial plexopathy or other mononeuropathies such as cubital tunnel syndrome or radial nerve palsy or carpal tunnel syndrome. However, it was not feasible. Furthermore, if neuropathy was found in the rhomboid muscle, the lesion was presumed to be cervical in origin. Second, we could not determine the severity of neuropathy. EMG can indicate the presence of neuropathy; however, further studies are necessary to evaluate its severity. Nevertheless, our study is the first to evaluate a large series of patients with RCTs using EMG. Third, we did not evaluate comorbidities such as diabetes or cerebrovascular disease, making it difficult to exclude their potential influence. Finally, we did not evaluate clinical outcomes, and therefore the effect of neuropathy on post-operative outcomes remains unclear. Further studies are necessary to clarify the influence of neuropathy on clinical outcomes. Despite these limitations, EMG should be considered selectively, particularly in large-to-massive tears, severe muscle atrophy, or pseudoparalysis, rather than as a routine test for all cases.

## Conclusion

Larger tear size was associated with a greater risk of concomitant neuropathy, including cervical spinal lesions. Furthermore, if RCTs coexist with neuropathy, fatty infiltration may worsen because of the combined effects of disuse atrophy due to RCTs and atrophy due to neuropathy. EMG evaluation may be particularly useful in cases of RCTs, especially large-to-massive RCTs.

## Disclaimers

Funding: No funding was disclosed by the authors.

Conflicts of interest: The authors, their immediate families, and any research foundations with which they are affiliated have not received any financial payments or other benefits from any commercial entity related to the subject of this article.
